# A Systematic Review of Non-Invasive Brain Stimulation Therapies and Cardiovascular Risk: Implications for the Treatment of Major Depressive Disorder

**DOI:** 10.3389/fpsyt.2012.00087

**Published:** 2012-10-10

**Authors:** Leonardo Augusto Negreiros Parente Capela Sampaio, Renerio Fraguas, Paulo Andrade Lotufo, Isabela Martins Benseñor, André Russowsky Brunoni

**Affiliations:** ^1^Department of Psychiatry, University of São Paulo Medical SchoolSão Paulo, Brazil; ^2^Clinical Research Center, University Hospital, University of São PauloSão Paulo, Brazil; ^3^Department of Internal Medicine, University of São Paulo Medical SchoolSão Paulo, Brazil; ^4^Department of Neurosciences and Behavior, Institute of Psychology, University of São PauloSão Paulo, Brazil

**Keywords:** transcranial direct current stimulation, major depressive disorder, repetitive transcranial magnetic stimulation, systematic review, heart rate variability, hypothalamo-hypophyseal system

## Abstract

Major depressive disorder (MDD) and cardiovascular diseases are intimately associated. Depression is an independent risk factor for mortality in cardiovascular samples. Neuroendocrine dysfunctions in MDD are related to an overactive hypothalamus-pituitary-adrenal (HPA) axis and increased sympathetic activity. Novel intervention strategies for MDD include the non-invasive brain stimulation (NIBS) techniques such as repetitive transcranial magnetic stimulation (rTMS) and transcranial direct current stimulation (tDCS). In fact, although these techniques have being increasingly used as a treatment for MDD, their cardiovascular effects were not sufficiently investigated, which would be important considering the dyad MDD/cardiovascular disorders. We investigated this issue through a systematic review for published articles from the first date available to May 2012 in MEDLINE and other databases, looking for main risk factors and surrogate markers for cardiovascular disease such as: cortisol, heart rate variability (HRV), alcohol, smoking, obesity, hypertension, glucose. We identified 37 articles (981 subjects) according to our eligibility criteria. Our main findings were that NIBS techniques might be effective strategies for down-regulating HPA activity and regulating food, alcohol, and cigarette consumption. NIBS’s effects on HRV and blood pressure presented mixed findings, with studies suggesting that HRV values can decrease or remain unchanged after NIBS, while one study found that rTMS increased blood pressure levels. Also, a single study showed that glucose levels decrease after tDCS. However, most studies tested the acute effects after one single session of rTMS/tDCS; therefore further studies are necessary to investigate whether NIBS modifies cardiovascular risk factors in the long-term. In fact, considering the burden of cardiac disease, further trials in cardiovascular, depressed, and non-depressed samples using NIBS should be performed.

## Introduction

Major depressive disorder (MDD) and cardiovascular disorders are both prevalent and disabling conditions. It is expected that, by 2020, they are going to be the second and the first main causes of disability, respectively, worldwide (Murray and Lopez, 1997). These disorders present a complex relationship, with one increasing the prevalence and severity of the other – e.g., subjects with depression have an increased risk for myocardial infarction (MI) while those with depression post-MI have increased risk of mortality (Taylor et al., 2005; Whooley et al., 2008). However, pharmacological treatments do not necessarily diminish cardiovascular risk, as shown in recent clinical trials in patients with cardiovascular disease and depression (Jiang et al., 2011). Further, tricyclic antidepressants and antiepileptic drugs (also used in some types of depression) decrease heart rate variability (HRV), which is a surrogate marker of cardiovascular illness (Kemp et al., 2010; Lotufo et al., 2012). Likewise, tricyclics might also increase levels of IL-6 and TNF-alpha that are pro-inflammatory cytokines associated with cardiovascular risk (Maes et al., 2011). Therefore, the development of treatments that are not only clinically effective but also safe from a cardiovascular perspective is needed.

In recent years, non-invasive brain stimulation (NIBS) techniques, such as repetitive transcranial magnetic stimulation (rTMS) and transcranial direct current stimulation (tDCS), have been investigated as putative treatments for depression. Transcranial magnetic stimulation (TMS) depolarizes neurons through a potent, relatively focal, electromagnetic field that is generated beneath a coil positioned over the scalp. When applied repetitively, rTMS has therapeutic effects. Recent meta-analyses demonstrated that high-frequency (HF) rTMS (i.e., excitatory) over the left dorsolateral prefrontal cortex (DLPFC) and low-frequency (LF) rTMS (i.e., inhibitory) over the right DLPFC are effective treatments for MDD (Schutter, 2009, 2010). In contrast to TMS, tDCS applies weak, direct electric currents to the brain through relatively large electrodes placed over the scalp (Brunoni et al., 2012a). For MDD, the anode is placed over the area corresponding to the left DLPFC. Recently, a meta-analysis (Kalu et al., 2012) and a large randomized, clinical trial (Brunoni et al., in press) suggested that tDCS might be an effective treatment for depression.

Therefore, considering that (1) cardiovascular disease and depression are related conditions; (2) antidepressants might have a hazardous impact in increasing cardiovascular risk and (3) NIBS therapies are potential therapeutic interventions for depression, we sought to systematically review the effects of such techniques on cardiovascular risk factors. We looked for risk factors such as increased blood pressure and serum glucose levels, obesity, alcohol use, and smoking. We also included in our search strategy HRV and hypercortisolism that are markers of cardiovascular risk. Importantly, we did not look specifically for studies with MDD, as we anticipated that the total number of studies would be low. Rather, our aim was to summarize current evidence of the effects of NIBS techniques on cardiovascular risk factors and discuss their implications in the treatment of MDD. Nevertheless, our findings can be generalized for other neuropsychiatric disorders in which pharmacological treatment is associated with hazardous cardiovascular effects.

## Materials and Methods

We performed a systematic review for published articles from the first date available to May 2012 in the following databases: MEDLINE, Scielo, and Web of Knowledge. According to our proposal, the key search terms were:

#1 “TMS” (MeSH term) OR “tDCS” OR (“transcranial” AND “stimulation”)
#2 “Hypothalamo-hypophyseal system” (MeSH term) OR “hypothalamus-pituitary-adrenal (HPA) axis” OR “cortisol”
#3 “HRV” OR “RR variability”
#4 “Smoking”
#5 “Obesity” (MeSH term) OR “food”
#6 [“Alcohol” AND (“dependence” OR “abuse”)] OR “alcohol”
#7 “Blood pressure” OR “hypertension”
#8 “Glucose” OR “diabetes”

The key search term #1 was crossed with (Boolean term AND) each other term. We included all original clinical studies written in English, apart from studies in experimental animals. We also examined reference lists in retrieved papers, and contacted experts on the field for identifying additional articles.

## Results

**Figure 1:**
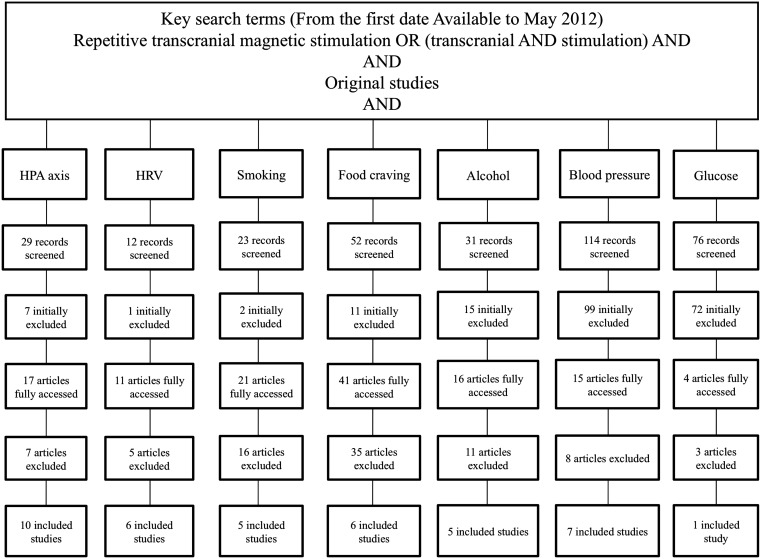
**Flow chart of reviewed studies, according to PRISMA guidelines**. The complete key search terms are described in the main text.

Figure 1 depicts the search strategy and included articles. Table 1 describes each article individually. We identified 37 articles, corresponding to 40 studies, since some articles reported more than one cardiovascular risk factor. We further discuss NIBS effects on each risk factor.

**Table 1:** **Summary of reviewed studies**.

	*N*	Sample	Protocol	Findings
**HYPOTHALAMIC-PITUITARY-ADRENAL (HPA) SYSTEM**				
Masur et al. (1991)	10	HS	HF-rTMS at F3	rTMS had no impact on cortisol levels
Evers et al. (2001)	23	HS	HF-rTMS at F3	rTMS mildly decreased cortisol levels
Zwanzger et al. (2003)	37	MDD	HF-rTMS at F3	rTMS did not affect cortisol levels after the DEX/CRH test
Zwanzger et al. (2007)	11	HS	LF-rTMS at F4	rTMS did not affect cortisol levels after panic induction with CCK-4
Baeken et al. (2009a)	20	MDD	HF-rTMS at F3	rTMS decreased cortisol immediately and after 30 min
Baeken et al. (2009b)	54	HS	HF-rTMS at F3 or F4	rTMS had no impact on salivary cortisol levels
Baeken et al. (2011)	24	HS	HF-rTMS at F4	Baseline anxiety modified cortisol expression after rTMS
Raimundo et al. (2012)	50	HS	atDCS at C3	rTMS had no impact on cortisol levels
Binkofski et al. (2011)	15	HS	atDCS at M3	atDCS decreased cortisol levels
Brunoni et al. (2012b)	20	HS	atDCS at F3/ctDCS at F4 and vice-versa	atDCS at F3 decreased cortisol levels
**HEART RATE VARIABILITY (HRV)**				
Yoshida et al. (2001)	32	HS	LF-rTMS at Cz	rTMS increased low-frequency HRV
Udupa et al. (2007)	52	MDD	HF-rTMS at F3	rTMS improved sympathovagal balance
Goldie et al. (2010)	6	HS	Unclear	HRV effectively measures arousal levels
Vandermeeren et al. (2010)	30	HS	atDCS or ctDCS at Fz	No differences in HRV between groups
Montenegro et al. (2011)	20	HS	atDCS at T3	Sympathovagal balance decreased only in athletes
Brunoni et al. (2012b)	20	HS	aTDCS at F3/ctDCS at F4 and vice-versa	atDCS at F3 increased high-frequency HRV during negative imagery
**BLOOD PRESSURE (BP)**				
Jahanshahi et al. (1997)	6	HS	HF-rTMS at M3	HF-rTMS showed a trend to decrease BP (*p* = 0.06)
Jenkins et al. (2002)	19	HS	LF-rTMS at F3 or F4	Left but not right rTMS decreased BP
Yozbatiran et al. (2009)	12	Stroke	HF-rTMS at M1	rTMS increased BP
Vandermeeren et al. (2010)	30	HS	atDCS at Fz	No differences in BP between groups
Van den Eynde et al. (2011)	38	BN	HF-rTMS at F3	No effects in BP between groups
Binkofski et al. (2011)	15	HS	atDCS at M3	tDCS decreased BP
Knotkova et al. (2012)	8	MDD	aTDCS at F3	BP initially increased and then decreased after tDCS
**GLUCOSE LEVELS**				
Binkofski et al. (2011)	15	HS	atDCS at M3	tDCS increased glucose tolerance
**FOOD INTAKE**				
Uher et al. (2005)	28	HS	HF-rTMS at F3	Food craving increased after sham, but not after real stimulation
Fregni et al. (2008b)	23	HS	aTDCS at F3/ctDCS at F4 and vice-versa	tDCS decreased food craving
Camus et al. (2009)	56	HS	HF-rTMS at F4	rTMS decreased increased evaluation for food
Van den Eynde et al. (2010)	38	BN	HF-rTMS at F3	rTMS decreased urge to eat and binge-eating episodes
Barth et al. (2011)	10	HS	HF-rTMS at F3	Prefrontal rTMS inhibited food cravings no better than sham rTMS
Goldman et al. (2011)	19	HS	atDCS at F4/ctDCS at F3	tDCS decreased food craving
**NICOTINE DEPENDENCE**				
Eichhammer et al. (2003)	14	Smokers	HF-rTMS at F3	rTMS reduced cigarette smoking
Fregni et al. (2008a)	24	Smokers	atDCS at F3 or F4	Left or right tDCS reduced craving
Amiaz et al. (2009)	48	Smokers	HF-rTMS at F3	rTMS reduced cigarette consumption and craving
Boggio et al. (2009)	27	Smokers	atDCS at F3	tDCS decreased craving after smoking cue exposure
Rose et al. (2011)	15	Smokers	HF-rTMS and LF-rTMS at SFG	Differential effects of craving according to type of stimulation
**ALCOHOL DEPENDENCE (AD)**				
Boggio et al. (2008)	13	AD	aTDCS at F3/ctDCS at F4	tDCS decreased alcohol craving
Mishra et al. (2010)	45	AD	HF-rTMS at F4	rTMS had significant anticraving effects in alcohol dependence
Höppner et al. (2011)	19	AD	HF-rTMS at F3	No differences in craving and mood between groups
Herremans et al. (2012)	36	AD	HF-rTMS at F4	No significant effects on alcohol craving
Nakamura-Palacios et al. (2012)	49	AD	HF-rTMS at F3	tDCS increased frontal activity for Lesch IV alcoholics

*HS, healthy subjects; MDD, major depressive disorder; BN, bulimia nervosa; AD, alcohol dependence; HF- and LF-rTMS, high-frequency and low-frequency repetitive transcranial magnetic stimulation, respectively; atDCS, anodal transcranial direct current stimulation; ctDCS, cathodal transcranial direct current stimulation; BP, blood pressure; HRV, heart rate variability; F3 and F4, left and right dorsolateral prefrontal cortical area; M3, left motor cortical area; T3, left temporal area; SFG, superior frontal gyrus*.

### HPA axis

Masur et al. (1991) evaluated cortisol plasma levels in healthy volunteers, observing no specific rTMS effects. Later, Evers et al. (2001) evaluated healthy subjects in a sham-controlled design, finding that rTMS decreased cortisol serum levels; whereas Baeken et al. (2009b) performed a sham-controlled study in healthy women, finding no rTMS effects on the HPA axis. However, Baeken et al. (2011) found, in another sample, that rTMS decreased cortisol levels when controlling for baseline state anxiety. Other studies reported that rTMS decreased salivary cortisol levels in patients with depression (Baeken et al., 2009a) and bulimia (Claudino et al., 2010). Zwanzger et al. (2003, 2007) evaluated rTMS effects on the “area under the curve” of cortisol response in two studies: in depressed patients, the authors explored whether therapeutical rTMS would change the cortisol response to the dexamethasone cortisol suppression test that is characteristically impaired in depression, finding no rTMS effects. Later on, the authors evaluated whether rTMS would decrease panic symptomatology and cortisol response after experimentally induction of a panic attack with CCK-4. They also found no specific rTMS effects.

Raimundo et al. (2012) evaluated tDCS effects on cortisol plasma levels in healthy volunteers, with no specific effects. Conversely, Binkofski et al. (2011) observed that anodal tDCS decreased cortisol serum levels compared to sham. Finally, Brunoni et al. (2012b) also found cortisol levels decreasing after anodal tDCS coupled with negative imagery viewing, which is a method to experimentally induce stress.

### Heart rate variability

Yoshida et al. (2001) suggested that rTMS could activate the sympathetic nervous system as LF power increased – however, LF reflects both sympathetic and parasympathetic activity (ESC, 1996) and therefore a more appropriate conclusion would be that rTMS activated both the sympathetic and parasympathetic systems. Udupa et al. (2007), in a randomized, controlled trial compared HRV changes after escitalopram and rTMS in depressed subjects, suggesting that rTMS not only improved depression but also restored sympathovagal balance (i.e., increased HRV values), in contrast with escitalopram. Finally, Goldie et al. (2010) described that rTMS led to “decreased arousal levels” (i.e., decreased sympathetic/increased parasympathetic activity), although the study primary aim was to identify novel HRV measures, and not rTMS effects on HRV *per se*.

Regarding tDCS, Vandermeeren et al. (2010) applied anodal, cathodal, and sham tDCS in 30 healthy volunteers, finding no specific effects on HRV. Conversely, Montenegro et al. (2011) applied anodal tDCS over the left temporal area, observing HF HRV increasing and LF-HRV decreasing; however, this effect was observed only in highly fit athletes. Finally, Brunoni et al. (2012b) found that anodal tDCS increased HF-HRV, particularly during negative imagery viewing, suggesting that tDCS could interfere favorably in stress responses elicited by negative imagery.

### Blood pressure

In a safety study, Jahanshahi et al. (1997) evaluated HF rTMS in six healthy subjects, finding a trend (*p* = 0.06) for active rTMS decreasing blood pressure. Jenkins et al. (2002) compared the effects of rTMS on mood and blood pressure over the left and right DLPFC finding that left but not right stimulation decreased arterial pressure. Likewise, Knotkova et al. (2012), in an open-label study, found that anodal tDCS over the left DLPFC generally decreased blood pressure levels. In addition, Binkofski et al. (2011) used anodal tDCS over the left primary motor cortex, finding small but significant decrements on blood pressure.

Conversely, Yozbatiran et al. (2009) found a small but significant *increase* of systolic blood pressure after HF rTMS stimulation over the ipsilesional primary motor cortex hand area in stroke patients. Other studies with rTMS (Van den Eynde et al., 2010) and tDCS (Vandermeeren et al., 2010) showed no changes in blood pressure.

### Serum glucose levels

Here, no rTMS studies matching our eligibility criteria were found. For tDCS, Binkofski et al. (2011) studied male healthy volunteers after active and sham tDCS stimulation. They observed increased systemic glucose uptake tolerance in the active group.

### Food intake

Fregni et al. (2008b) found that desire for food consumption and visual attention for food imagery decreased after tDCS, in a sham-controlled design with healthy volunteers. Similar findings were demonstrated in subsequent tDCS (Goldman et al., 2011) and rTMS (Camus et al., 2009; Van den Eynde et al., 2010) studies. Uher et al. (2005) found that self-reported food craving during exposure to food remained stable before and after active rTMS, while it increased after sham stimulation. However, Barth et al. (2011) found that rTMS inhibited food craving no better than sham rTMS.

### Smoking

Fregni et al. (2008a) compared active vs. sham tDCS over the left DLPFC in smokers and found specific tDCS effects in decreasing cue-induced craving. In fact, another study showed that such effects were cumulative over 5 days (Boggio et al., 2009). Amiaz et al. (2009) reported similar findings, and also that rTMS decreased cigarette consumption. Conversely, Eichhammer et al. (2003) reported that rTMS decreased cigarette smoking although not levels of craving. Finally, Rose et al. (2011) found that HF-rTMS over the superior frontal gyrus increased craving after smoking cue presentations, while it decreased craving after neutral cue ones.

### Alcohol consumption

Herremans et al. (2012) showed no rTMS effects on alcohol craving in alcohol dependent patients. Conversely, Mishra et al. (2010) reported positive findings for rTMS in reducing alcohol craving. Höppner et al. (2011) showed mixed findings: although rTMS had no effect on alcohol craving, the authors found that real vs. sham rTMS increased the attentional blink on alcohol-related pictures.

Regarding tDCS, Boggio et al. (2008) found that active tDCS decreased alcohol craving in patients with alcohol dependence. Further, Nakamura-Palacios et al. (2012) found that active vs. sham tDCS increased cognitive performance and prefrontal activity in Lesch IV alcoholics.

## Discussion

In this systematic review, we evaluated 37 studies (981 subjects) addressing the effects of NIBS techniques on several risk factors. We found that: (1) cortisol levels decrease or remain unchanged after NIBS; (2) NIBS effects on HRV and blood pressure presented mixed findings, with studies suggesting that HRV values can decrease or remain unchanged after NIBS (one study found that rTMS increased blood pressure levels); (3) a single study showed that glucose levels decrease after tDCS; (4) most studies found that NIBS can effectively decrease craving for alcohol, smoking, and food intake. We further discuss these findings.

First, some limitations should be underscored. Although the studies generally presented adequate methodology in terms of design and assessment, most of them explored the effects of NIBS after one single session. In addition, most studies were exploratory, without a clearly defined hypothesis. Therefore, the evidence here presented should be considered preliminary and hypothesis-driven for further studies.

The effects of NIBS on the HPA axis and cardiovascular mechanisms are likely to occur via a top-down modulation, i.e., changes in cortical activity that subsequently modify the activity of centers related to hormonal and cardiovascular regulation that are situated in subcortical areas and in the brain stem. Of note, NIBS effects on these systems occurred mostly in the context of stress response – e.g., negative image visualization (Brunoni et al., 2012b) and 16 h fasting (Binkofski et al., 2011) or in clinical samples, such as depression (Baeken et al., 2009a), bulimia (Claudino et al., 2010), and anxiety (Baeken et al., 2011). Likewise, such effects occurred primarily during neuromodulation of DLPFC, an area implicated in the pathophysiology of stress and affective disorders (Koenigs and Grafman, 2009). This suggests that NIBS effects might not act on these systems during their steady state; but rather when they are being activated – for instance, during a stress response. Further studies should investigate in which conditions blood pressure and cortisol expression can be modulated by NIBS; and also which brain areas should be targeted. In this context, using computer simulated models and/or neuroimaging studies to identify which brain areas are activated during brain stimulation might help to identify cortical areas that could be targeted in order to indirectly modulate the subcortical and brain stem areas related to cardiovascular control. Another option is to investigate the use of novel NIBS techniques, such as “deep rTMS” (Levkovitz et al., 2007) and “high-definition” tDCS (Minhas et al., 2010) that can theoretically target deeper brain areas and/or induce more focalized stimuli as to directly modulate those areas.

Most reviewed studies showed that NIBS effectively regulated craving and consumption for food, alcohol, and cigarette smoking. This is in agreement with the putative role of the prefrontal cortex as a brain region related to decision-making, either of non-emotional (“cold cognition”) and emotional (“hot cognition”) content (Koenigs and Grafman, 2009), the latter including also the inhibitory control of appetitive impulses. This brain area is also connected with subcortical areas related to the “reward system,” which includes the ventral tegmental area and nucleus accumbens (Fecteau et al., 2010). Modulation of food, alcohol, and cigarette intake could be a crucial contribution of NIBS for the cardiovascular field, since most risk factors are directly associated with poor dietary and lifestyle choices. However, since most studies evaluated the acute effects of tDCS, further investigation should focus on the long-term effects of tDCS in regulating craving.

Finally, Binkofski et al. (2011) observed increased systemic glucose turnover with active tDCS. This is a proxy for insulin resistance that is impaired in diabetes mellitus. The authors suggested that their findings could be explained by the activation of ATP-dependent potassium channels (KATP) by tDCS in the hypothalamus, which would inhibit hepatic gluconeogenesis. Further studies could evaluate whether tDCS modifies other mechanisms of insulin metabolism in healthy and clinical samples.

### Implications for MDD treatment

The activation of the HPA axis is an important link between MDD and cardiovascular illness (Maes et al., 2011). Hypercortisolism leads to a dysregulation of glucose, lipid metabolism, and blood pressure, which contributes to the metabolic syndrome. Moreover, hypercortisolism has unfavorable effects on the immune system. In our review, we observed that some NIBS studies decreased cortisol levels, suggesting that these techniques might induce modulatory top-down effects that lead to a down-regulation of the HPA axis. Further research should explore whether such effects persist over time – for instance, evaluating other cortisol measures, such as cortisol awakening response and cortisol daily curve.

Major depressive disorder is also related to an autonomic shift toward sympathetic predominance, which also increases inflammatory response in the endothelium and platelet activation, hastening the formation of atherosclerotic plaques (Lett et al., 2004). This sympathetic tone shift boosts sympatho-adrenal response, increasing catecholamines secretion in adrenal medulla, leading to a hypercortisolemic and hypercatecholaminergic state, contributing to this physiopathological mechanism. Our results showed that HRV is either non-affected or increased after NIBS (Evers et al., 2001). A possible mechanism of action for NIBS is also top-down modulation, through activity increasing in cortical regions and down-regulation of the sympatho-adrenomedullary system, leading to a decrease in sympathetic activity.

### Other brain stimulation techniques

Although NIBS seems to down-regulate stress response, this might not necessarily be true to other forms of brain stimulation. For instance, electroconvulsive therapy (ECT) has a characteristic biphasic response, i.e., a parasympathetic phase that lasts for approximately 20 s followed by a sympathetic phase of approximately 1 min (Geersing et al., 2011). In addition, repetitive trans-spinal magnetic stimulation, i.e., a TMS coil applied over the sixth and seventh cervical vertebrae, showed no difference between sham and active groups on measures of HRV (Paxton et al., 2011). However, deep brain stimulation seems to have favorable effects over the autonomic nervous system in patients with Parkinson’s disease, in contrast to levodopa that has unfavorable effects (Ludwig et al., 2007).

Another potentially interesting therapy is vagus nerve stimulation (VNS). Recently, a HRV meta-analysis of epilepsy and epilepsy treatment suggested that could have favorable effects on sympathovagal balance, presumably through direct stimulation of the vagus nerve (Lotufo et al., 2012). Further, it also seems to be a safe therapy from a cardiovascular perspective, according to a recent long-term follow-up in depressed patients (Bajbouj et al., 2010).

## Conclusion

We found that NIBS techniques, used in neuropsychology and neuropsychiatry, might also exert effects in pathological endocrine, immune, and neural events associated with cardiovascular disease, particularly in managing the cardiovascular reactivity induced by stress and inhibiting food and cigarette consumption. For MDD, these techniques contrast with antidepressants not only due to the absence of pharmacological interaction, but also possibly in terms of cardiovascular effects. Nevertheless, further studies are needed to determine the scope of these interventions, defining their effect power and indications for regulating cardiovascular and metabolic parameters.

## Conflict of Interest Statement

The authors declare that the research was conducted in the absence of any commercial or financial relationships that could be construed as a potential conflict of interest.
